# Ecological and Geographical Structure of the Plant Cover of the East Asian Boreal–Nemoral Ecotone (the Lower Amur Region, Far East Russia)

**DOI:** 10.3390/plants12030615

**Published:** 2023-01-30

**Authors:** Maria V. Kryukova

**Affiliations:** Institute of Water and Ecology Problems FEB RAS, Khabarovsk Federal Research Center FEB RAS, 56 Dikopoltseva, 680000 Khabarovsk, Russia; flora@ivep.as.khb.ru; Tel.: +7-(4212)-227573

**Keywords:** species, genera, family, taxonomic spectrum, floristic complex, spatial structure of the plant cover

## Abstract

The study of the biodiversity of vegetation cover in the context of its genesis and development is an important task. The results of these studies are the basis for the development of ecological, biogeographical, evolutionary, and sociological research, such as modelling the dynamic processes of natural ecosystems, understanding the consequences of natural and anthropogenic changes for biodiversity, solving problems of biodiversity conservation, etc. Of particular interest from this point of view is the biodiversity of ecotones, which can serve as a model for studying the factors of the genesis of the plant cover structure in a dynamic environment. In this paper, we analyze the taxonomic structure of the flora of vascular plants and the spatial structure of the plant cover in the East Asian boreal–nemoral ecotone (of the Lower Amur region). The botanical research was conducted through the application of traditional techniques for floristic and geobotanical studies. The material for this article was drawn from over 15,000 herbarium samples and 1400 floristic and geobotanical descriptions made between 1993 and 2021 in the Lower Amur region. The analyzed flora includes 2240 species from 760 genera and 158 families, which constitute 80% of the species composition of the Russian part of the Amur River basin. The native flora comprises 1801 species from 602 genera and 152 families. The species diversity and quantitative characteristics of the natural and adventive flora of vascular plants in the Lower Amur region are comparable to those of the southern limit of the distribution of taiga ecosystems in the Holarctic. The spectrum of the leading families and genera in terms of the number of species corresponds to the geographical position of the territory (the family spectrum is led by *Asteraceae*, *Cyperaceae*, *Poaceae*, *Ranunculaceae*, *Rosaceae* and *Polygonaceae* and in the generic spectrum, *Carex*, *Artemisia*, *Salix*, *Viola*, *Saxifraga*, *Poa* and *Saussurea*). The specificity of the flora is determined by a combination of elements in the boreal and sub-boreal flora of East Asia. Seven floristic complexes are defined for the Lower Amur region flora: forest (41.4% of the native flora), meadow (19.2%), mire (4.1%), mountain tundra (12.5%), rocky scree (8.9%), aquatic–semiaquatic (7.8%) and floodplain–estuarine shallow (6.2%). The regularity of some floristic complexes is defined by the landscape’s ecological conditions, and the variety in the edaphic, orographic and climatic parameters within the region. The spatial structure of the plant cover of the boreal–nemoral ecotone is described.

## 1. Introduction

Understanding the natural and anthropogenic mechanisms of changes in species diversity and the structure of vegetation cover in space and time is the basis of biological, biogeographical, ecological, evolutionary, and sociological studies. From this point of view, the contact zones of various plant formations act as an important source of information about the processes that define the formation and maintenance of biological diversity, and can be considered a model for studying, monitoring and predicting the response of biological diversity of vegetation cover to natural and anthropogenic changes in the natural environment [1,2,3,4,5]. This gives rise to the interest in the vegetation cover of the monsoonal region of the Amur basin, which is a unique object of study when addressing the factors and mechanisms related to the formation of regional vegetation cover structure of ecotone territories. Furthermore, the Lower Amur region is an arena of intensive economic development. The conservation of biological and landscape diversity, and the ecological functions of vegetation in these conditions, are very important problems that cannot be solved without addressing the vegetation cover, the history of its formation and development, its contemporary structure, and the consequences of anthropogenic impacts. Additionally, the mechanisms of phytogenic stability of landscapes need to be identified, since the vegetation cover is one of the main structural and functional components of ecosystems that react sharply to changes in natural conditions.

The Lower Amur basin covers a large territory between 45°–56° N, 131°–141° E. The total length of this territory from south to north is more than 700 km, and in the latitudinal direction it extends up to 500 km. The natural boundaries of this region are mountains—Malyi Khingan, Bureinskyi, Dusse Alin, Yam Alin, Magu, Mevachan and Omalskyi ridges in the west and northwest, and the mountain system of Sikhote–Alin in the east.

This territory is located on the eastern side of Asia in the zone of active interaction between the mainland and the Pacific Ocean, which gives rise to the high contrast between natural and climatic conditions, and the combination of several natural boundaries (geological, geomorphological, zonal–climatic, etc.), which determine the contact zones of boreal and nemoral phratries of plant formations of different genesis (Okhotsko–Kamchatense, Amurense, Angaridense, Siberian and Beringian) [6,7,8].

The mountain–taiga landscape with middle and lowland relief and significant areas of intermountain flatlands characterize the Lower Amur region. The hydrographic network of the territory is quite well-defined; the main water artery is the Amur River. On the plains, the Amur channel branches strongly, forming complex systems of channels, branches and reservoirs. The flat territories contain sections of the lower reaches of several large tributaries of the Amur River: Tunguska, Ussuri, Anyui, Gura, Amgun and some other watercourses.

Concerning the climatic zoning of Khabarovsk Krai, the territory under consideration is located at the junction of the Middle Amur, Badzhalsko–Bureya, Sikhote–Alin and Amgun–Lower Amur provinces of the monsoon forest climatic region [9]. The provincial boundaries, determined by the transition in the ratio of heat and moisture through a certain gradient, cause fundamental changes in the structure and function of natural ecosystems of the zonal type. The warmest thermal regions are characterized by maximum average daily air temperatures above 10 °C, exceeding 2400 °C per year. These areas are located within the Middle Amur lowland and are the most favorable for the development of thermophilic sub-boreal and sub-boreal–subtropical species. Cool and even moderately cold climatic zones are located in the north of the study area. Within the Evoron–Chukchagir and Amgun–Amur lowlands, the sums of temperatures above 10 °C decrease to 1600–1700 °C per year [9].

Specific natural and climatic conditions of the Amur basin comprise cyclic long-term changes in dry and humid periods, varying degrees of oceanic and continental influence in the east and west of the basin, and the presence of mountain systems located perpendicular to the monsoonal movement of air masses. These determine a high contrast of factors and a combination of high-ranking natural boundaries.

The Amur River Valley (within 51–52° N) is the main frontier for the distribution of thermophilic East Asian sub-boreal and sub-boreal–subtropical species. Severe natural and climatic conditions, together with the proximity of foci of permafrost in the north of the study area, prevent their widespread establishment in the Amur region. The presence of significant swampy areas in the valleys of the lower reaches of the Amur and Amgun rivers, along with submeridional-trending mountain systems, contribute to the penetration of hypoarctomontane, arcto-alpine and arcto-boreal elements (*Salix rhamnifolia* Pall., *S. polaris* Wahlenb., *S. fuscescens* Andersson, *Carex brunnescens* (Pers.) Poir., *C. rariflora* (Wahlenb.) Sm.), bringing together the floras of the western Okhotsk region and the northern part of the Lower Amur region.

The Lower Amur region has long attracted the attention of researchers, and the literature on the flora and vegetation of this region is also extensive. A detailed analysis of these studies was performed by Kozhevnikov and Kozhevnikova [10] and Kryukova [11]. Some studies address research on the species diversity of various territories belonging to the Lower Amur region ([10,12,13,14,15,16,17,18,19,20,21,22,23], etc.), or some taxonomy groups [24,25]. Analysis of the genesis and structure of some forest, meadow and mire plant formations is given in some studies ([6,7,8,26,27,28,29,30,31,32,33], etc.). At the same time, despite the almost 200-year history of studying the vegetation cover of the Amur River basin, there are no comprehensive works devoted to the composition and analysis of the vegetation cover of this territory. Existing botanical reports on the Far East region, the Khabarovsk Territory, or individual local territories related to the territory under consideration are either outdated or do not fully reflect the current state of the vegetation cover in the Lower Amur region. Owing to the uneven dispersal of knowledge, the task of generalizing and analyzing all information on the flora of this territory is very relevant.

The current research aimed to inventory the species diversity and perform a taxonomic analysis of the flora of vascular plants, so as to identify zonal and regional patterns in the spatial structure of the vegetation cover under the conditions of the zonal–climatic ecotone.

These data have mainly theoretical value for the study of the processes of the vegetation cover’s evolution in the southern Far East under the conditions of natural and anthropogenic environmental dynamics. They are also of practical interest for use as a basis for monitoring the dynamics of species diversity, the structural changes in vegetation cover, assessing the resistance of vegetation cover to natural and anthropogenic factors, and modelling the succession processes when these factors change.

## 2. Results

### 2.1. Taxonomic Structure

The flora of the Lower Amur region includes 2240 species from 760 genera and 158 families, which constitutes 80% of the species composition within the Russian part of the Amur River basin [10,11]. The native flora comprises 1801 species from 602 genera and 152 families. The adventive complex is represented by 439 species from 256 genera and 57 families, which is 19.6% of the total flora.

An impression of the features of zonal changes in the systematic composition of the flora of the territory, together with the main directions of florogenesis, is given by analysis at the level of families, genera and species. Table 1 shows the head spectrum of the largest families in terms of the number of species in the Lower Amur region. The ten largest families in the family–species spectrum contain 935 species, which is more than half of the entire flora (51.8%). The composition of the families in the first triad of the native flora of vascular plants indicates that they belong to arcto-boreal–East Asian flora.

In the flora of the Lower Amur region, as elsewhere in various areas of the Holarctic, the family *Asteraceae* dominates with 185 species, which is 10.3% of the total number of flora [34,35,36,37,38,39]. The absolute and relative diversity of this family is clearly expressed in different parts of the territory—in the southern plain, they differ due to the diversity of the species and genera of the Amur forest floristic complex of broadleaf forests, petrophilius rocky scree, rocky coeno-elements and floodplain meadow complex. On the massive uplifts in the northern half of the Lower Amur region, located in the suboceanic sector of the Pacific Ocean, the role of the family is somewhat reduced. It gives way to the family *Cyperaceae*, which achieves the highest rates due to the species and generic diversity of representatives of the mountain tundra floristic complex.

The families *Cyperaceae* and *Poaceae*, comprising 172 (9.6%) and 154 taxa (8.4%), respectively, are represented by a set of specific species in mountain systems, in forests and meadow formations of the Amur Valley. The species richness of the family *Cyperaceae* is mainly associated with the diversity of the genus *Carex*, whose representatives have mastered various ecotopes of the Amur River basin, from the plains and sea coast to the subalpine and alpine belts of mountain systems framing the river valley. In the southern plain areas of the Amur region, the generic diversity of the family *Cyperaceae* increases due to the presence of the genera *Rhynchospora*, *Fimbristylis* and *Cyperus*, and in the northern mountain systems, *Eriophorum*, *Carex*, etc., are present. Representatives of the *Poaceae* family, which occupies third place in terms of richness and diversity, grow in open habitats under the conditions of river floodplains, sea coasts and mountain tundra, as well as in forest communities under various conditions, and in some cases act as coenose-formers. Species of the genus *Calamagrostis* form floodplain reed grass, reed-forb and sedge-reed grass meadows on vast expanses of flat territories, while species of *Poa* grow on stony outcrops and scree, *Ptilagrostis* and *Trisetum* grow in mountain tundra groups, and *Glyceria*, *Phragmites*, *Zizania* and *Eragrostis* grow along the banks of rivers and lakes.

According to the composition of the second triad of families, the floras differ somewhat. The emergence of the *Ranunculaceae* family in the fourth position determines the assignment of the native flora to the *Ranunculaceae* subtype, i.e., to meadow and mountain floras. The leading positions of the families *Polygonaceae*, *Caryophyllaceae*, *Lamiaceae*, *Fabaceae*, *Scrophulariaceae* and *Orchidaceae*, with minor changes in ranks, imply the flora of the region have forest humid features. We should emphasize the *Polygonaceae* family, which occupies the sixth place in the ranking of families of native flora, and is a specific feature of the Russian part of the Amur basin, previously noted [10] as the defining “Amur”, gravitating toward the subtropical subtype, a flora variant in that appears in the series of floras of the Holarctic. This is due to the predominance of the genera *Persicaria*, *Truellum* and *Fallopia* in this family, the centers of species diversity of which are located in the subtropical regions of East Asia.

Families represented in the flora by one species each (40 (2.2% of the flora of the Lower Amur region)), two or three species (36 (4.9%)) and four or five species (22 (5.2%)) indicate a significant number of families with few species and genera. This phenomenon is explained by the fact that a significant number of them are relict. The most ancient groups of vascular plants are represented in modern flora by the monotypic and oligotypic families *Nelumbonaceae*, *Adiantaceae*, *Cabombaceae*, *Phrymaceae*, *Salviniaceae*, *Taxaceae*, *Trapellaceae*, *Penthoraceae*, etc. Of great importance are also the manifestations of “edge effects”, which are associated with a natural decrease in participation by representatives of various florocenogenetic complexes at the boundaries of their distribution within the zonal climatic ecotone. The main diversity of the families *Schisandraceae*, *Chloranthaceae*, *Smilacaceae*, *Aristolochiaceae*, *Actinidiaceae* and *Dioscoreaceae* occurs in the subtropical and tropical regions of East Asia, and the families *Empetraceae*, *Diapensiaceae*, *Parnassiaceae* and *Tofieldiaceae* appear in cold and moderately cold, mainly mountainous regions of Asia and North America. In the flora composition of the Lower Amur region, they are each represented by one or two (or rarely three) species.

In total, 602 genera were identified in the flora of the Lower Amur region. The species diversity of the largest genera of flora is presented in Table 2. An analysis of the generic spectrum of the Lower Amur flora indicates an uneven distribution of species among flora genera. The ten leading genera of flora comprise 335 species, or 18.7% of the entire flora.

The ratio between the abundance of species and genera can serve as an indicator of florogenetic trends [40]. The high species saturation of genera is interpreted as a manifestation of autochthonous tendencies in the development of the flora. For the flora of the Lower Amur region, the ratio of the numbers of species to genera is 2.9:1, which may indicate the predominance of allochthonous processes in the development of flora over autochthonous ones. The poverty of the species diversity of genera and families is explained by the complexity of the processes of flora formation, in which a significant role is played by the change in the physical and geographical situation at the end of the Pliocene–Pleistocene. The immigration of species during a climatic change in different geological periods, in addition to the topographical diversity of the region, is an explanation for the plant diversity in the area. Only some genera show evidence of speciation within the Lower Amur region. These include the genera *Carex*, *Saxifraga*, *Salix*, *Deschampsis*, *Ptilagrostis*, *Saussurea* and others [23,24,25].

### 2.2. Ecological–Coenotical Structure of the Flora

**Figure 1:**
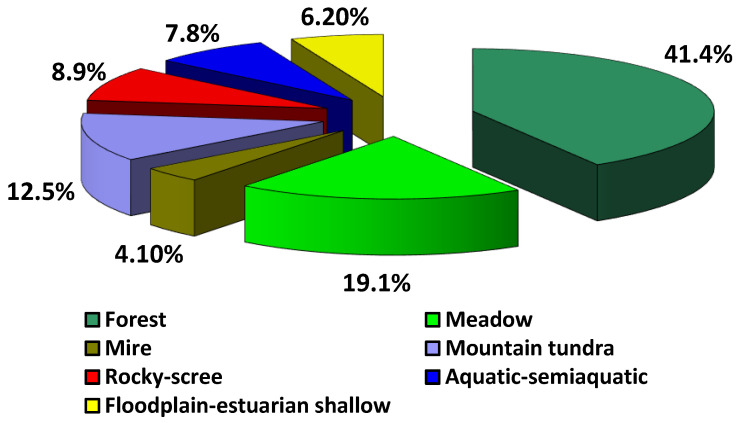
Spectrum of floristic complexes in the Lower Amir region.

The variety in adaptive capabilities of the flora species in the Lower Amur region determines the system of floristic complexes formed by more or less homogeneous groups of species that are similar in genetic or ecological–geographical features [41]. The existing scheme of floristic complexes proposed by L.I. Malyshev and G.A. Peshkova for Baikal Siberia was refined by A.P. Khokhryakov [42], A.E. Kozhevnikov [43], and V.Yu. Barkalov [44] when analyzing the flora of the Far Eastern region. Floristic complexes unite ecological–coenotic groups and elements confined to communities of various types according to the totality of environmental factors within ecotopes [45,46,47]. In the flora of the Lower Amur region, seven floristic complexes have been identified, the ratio of species diversity within which is shown in Figure 1.

#### 2.2.1. Forest Floristic Complex

Zoning on the territory of the Lower Amur region is characteristic of the forest floristic complex, since forests are predominant in terms of area, and fully comply with their modern climatic conditions. This complex comprises 745 species, or 41.4% of the entire flora. The leading families in terms of the number of species are *Asteraceae* (74 species), *Cyperaceae* (51), *Ranunculaceae* (48), *Rosaceae* (47), *Poaceae* (37), *Orchidaceae* (32), *Apiaceae* (21), *Salicaceae*, *Fabaceae* (19), *Violaceae* (18), *Convallariaceae* and *Lamiaceae* (16).

**Figure 2:**
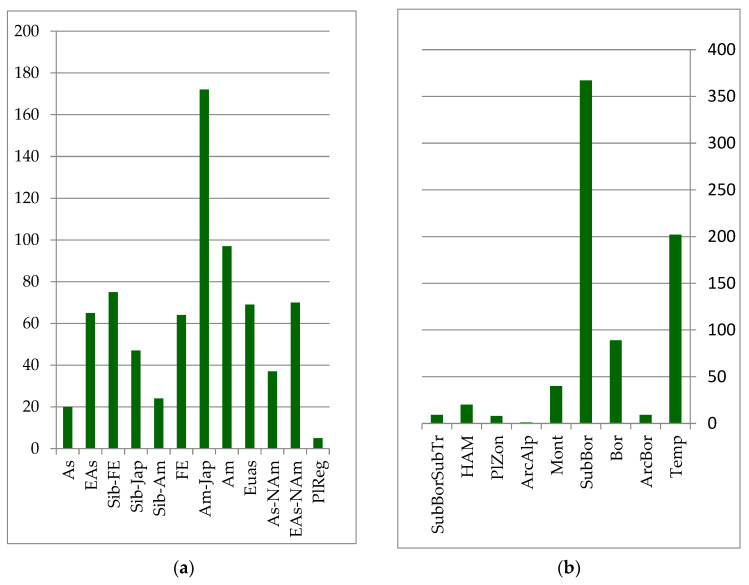
Number of species groups of the forest floristics complex: (**a**) longitudinal species groups: Asian (As); Eastern Asian (EAs); Siberian–Far Eastern (Sib-FE); Siberian–Japanese (Sib-Jap); Siberian–Amurense (Sib-Am); Far Eastern (FE); Amurense–Japanese (Am-Jap); Amurense (Am); Eurasian (Eas); Asian–North American (EAs-Nam); Eurasian–North American (Eas-NAm); PlReg–pluriregional; (**b**) zonal species groups: temperate (Temp); arcto–boreal (ArcBor); boreal (Bor); sub-boreal (SubBor); montane (Mont); arcto–alpine (ArcAlp); plurizonal (PlZon); hypoarcto–montane (HAM); sub-boreal–sub-tropical (SubBorSubTr).

The core of the forest floristic complex is formed by species whose distribution is associated with East Asia. The primary edificatory plants of these forest formations are represented by plant species of the Far Eastern, Siberian–Far Eastern, and Siberian–Japanese areal groups (see Figure 2): *Abies nephrolepis* (Trautv.) Maxim., *Picea ajanensis* Fisch. ex Carrière, *Larix cajanderi* Mayr (*Larix gmelinii* var. *gmelinii* (Rupr.) Kuzen.), *Pinus koraiensis* Siebold et Zucc., *Betula costata* Trautv., *Acer mono* Maxim. (*Acer pictum* subsp. *mono* (Maxim.) H. Ohashi), *Ulmus japonica* (Rehd.) Sarg. (*Ulmus davidiana* var. *japonica* (Rehder) Nakai), *Populus suaveolens* Fisch ex Poit. et A. Vilm., *Fraxinus mandshurica* Rupr. and others. *Populus tremula* L., *Duschekia fruticosa* (Rupr.) Pouzar (*Alnus alnobetula* subsp, *fruticosa* (Rupr.) Raus), *Swida alba* (L.) Opiz (*Cornus alba* L.), *Padus avium* Mill. (*Prunus padus* L.), *Salix bebbiana* Sarg. and others are more widely distributed in the forests of the temperate zones of Eurasia and North America. They feature in the formation of secondary groups in habitats that are affected by pyrogenic or other anthropogenic impacts, as well as in intrazonal plant groups on river floodplains. These species include *Picea obovata* Ledeb. and *Pinus sylvestris* L., which rarely form independent stands, but often appear as part of dark coniferous fir–spruce and larch forests. 

The predominant zonal element in the forest complex is the temperature group, which includes species of the boreal taiga zone and the sub-boreal zone of coniferous–broadleaf and broadleaf forests. These are insignificant in forest communities for all of the mountain–montane and subarctic–montane zonal elements (see Figure 2).

Modern climatic conditions contribute to the development of ecological–coenotic groups of light coniferous and dark coniferous forests on mountain slopes and plains. In the light coniferous forests of the Lower Amur region, 522 species grow, of which 355 reach an ecological optimum in forest communities, and the others are representatives of the mire, mountain–tundra and meadow floristic complexes. The most common types of larch forests are swampy psychrophilic sphagnum, green moss–ledum, herbaceous and aulyakomnia–sphagnum larch forests, light forests covering significant flat areas of the Amur region, psychromesophilic shrubs, mesophilic herbaceous–shrub forests of mountain slopes containing nemoral elements, and psychrophilic herbaceous larch forests of river floodplains and streams.

Modern dark coniferous and the associated forest types cover a significant part of the Lower Amur basin. According to A.S. Karpenko [27], in the middle of the last century, these types covered as much as 53% of the territory under consideration. The species diversity of dark coniferous formations includes 469 species of the forest floristic complex, while representatives of the meadow, rocky-scree, mountain–tundra floristic complexes are also noted. The most typical are mesophilic fir–spruce forests of fern, grass, green moss mountain slopes, and fern–grass valleys of rivers and streams. The low-mountain and mid-mountain areas of the Northern Sikhote–Alin are also characterized by mesophilic and psychromesophilic shrub–green moss fir–spruce forests, with a predominance of *Rhodococcum vitis-idaea* (L.) Avror. (*Vaccinium vitis-idaea* L.), *Vaccinium axillare* Nakai (*Vaccinium ovalifolium* var. *ovalifolium* Sm.) and *Ledum maximum* (Nakai) A. Khokhr et Mazurenko (*Rhododendron tomentosum* Harmaja) in the grass–shrub cover.

On the spurs of the Northern Sikhote–Alin, the southeastern part of the Bureya highlands, and in the contact zone of coniferous–broadleaf and dark coniferous taiga flora, nemoral species present in the cover and undergrowth play a significant part in the formation of spruce forests, with negligible participation in the composition of the main layer of forest stands *Pinus koraiensis*, *Betula costata*, *Acer tegmentosum* Maxim., etc. In contrast to the taiga green moss, small herb green moss and shrub green moss forests, these forests in the Lower Amur region are mainly associated with flat habitats.

In the southern part of the territory on mountain slopes, in the foothills, on the bottoms of river valleys and ancient river terraces, heat-loving formations of nemoral mesophilic and xeromesophilic Korean pine, Korean pine–broadleaf forests, and mixed psychromesophilic with participation by *Picea ajanensis*, *P. koraiensis* Nakai and *Abies nephrolepis* coniferous–broadleaf forests are widespread; on the rocky crests of watersheds, the tops of low mountains, steep slopes and gently sloping foothills, and fluvial heights in river floodplains, we see xeromesophilic and xerophilic oak forests formed by *Quercus mongolica* Fisch. ex Ledeb., with minor participation by *Betula davurica* Pall., *Acer mono*, *Tilia mandshurica* Rupr. et Maxim. and *T. amurensis* Rupr; on alluvial deposits in the valleys of small rivers and streams, we see mesophilic and migromesophilic elm–ash forests with *Ulmus japonica*, *U. laciniata* (Herder.) Mayr ex Schwapp., *Fraxinus mandshurica* and poplar forests with *Populus suaveolens*. These are the representatives of the nemoral forest ecological–coenotic group.

The species diversity of coniferous–deciduous and broadleaf forests includes 640 species of the forest floristic complex. Another 140 species grow in rocky scree habitats, along river banks as part of coastal–aquatic habitats, and in meadow communities, being protected under the canopy of these forest types. The cores of coniferous–broadleaf and broadleaf formations are species of the Amur and Amur–Japanese area groups (see Figure 2): *Pinus koraiensis, Tilia amurensis*, *Betula costata, Ulmus japonica* and many others. Near the northern limit of distribution, cedar–broadleaf forests grow only on mountain slopes, and are not found in river valleys, where climatic conditions are more severe due to temperature inversions.

Although the Korean pine–broadleaf and coniferous–broadleaf forests have decreased significantly in recent years, in some areas on the western spurs of the Sikhote–Alin, they have preserved their landscape-forming value. The diversity and complexity of the coniferous–broadleaf, Korean pine–broadleaf and broadleaf forests are determined by the quantitative richness of the tree, shrub and dwarf shrub–herb layers, along with the variety in the ecological and phylogenetic compositions of these elements. The humidity, groundwater level defining the degree of drainage of soils, and soil richness are of great importance to the distribution of tree layer edificators. The broadleaf tree species (*Tilia amurensis*, *T. taquetii*, *Ulmus laciniata*, *Betula costata*, *Juglans mandshurica* Maxim.) dominate on the wetter and richer soils of the gentle slopes and plateaus; *Pinus koraiensis* and *Quercus mongolica* dominate on soils with more drainage or that are temporarily moistened, and other coniferous and broadleaf tree species dominate in middling conditions (*Picea ajanensis*, *Abies nephrolepis*, *Salix abscondita* Laksch.).

The communities of these forests are rich in species, and most of them are unique East Asian relicts: *Panax ginseng* C.A. Mey., *Eleutherococcus sessiliflorus* (Rupr. et Maxim.) Maxim., *Asarum sieboldii* Miq., *Phyllitis japonica* Kom. (*Asplenium komarovii* Akasawa), etc. The valley of the Amur River at 50–52° N is the distribution boundary for over 90% of these thermophilic sub-boreal and sub-boreal–subtropical species. This boundary matches the main bioclimatic, geomorphological and landscape boundaries [9,48]. The ranges of these thermophilic species are elongated meridionally along the river, marking the main botanical and geographical ecotone “taiga–coniferous–broadleaf forests”.

The small areas of the relict species—*Taxus cuspidata* Siebold et Zucc., *Arisaema amurensis* Maxim., *Aralia elata* (Miq.) Seem., *Tilia amurensis*, etc.—are observed north of 51° N.

The ecological–coenotic groups of hygromesophilic floodplain small-leaf forests and stone birch forests, together with subalpine psychrophilic (alpine) creeping forests of cedar and alder dwarf, are slightly inferior in terms of total species richness (355, 226 and 132 species in the forest floristic complex, respectively).

#### 2.2.2. Meadow Floristic Complex

The meadow floristic complex comprises 345 taxa (19.1% of the total flora), while the species diversity is dominated by the families *Asteraceae* (58 species), *Poaceae* (56), *Cyperaceae* (25), *Polygonaceae, Ranunculaceae* (23), *Lamiaceae* (17), *Scrophulariaceae* (14)*, Fabacea* (13), *Rosaceae* (12) and *Juncaceae* (9).

**Figure 3:**
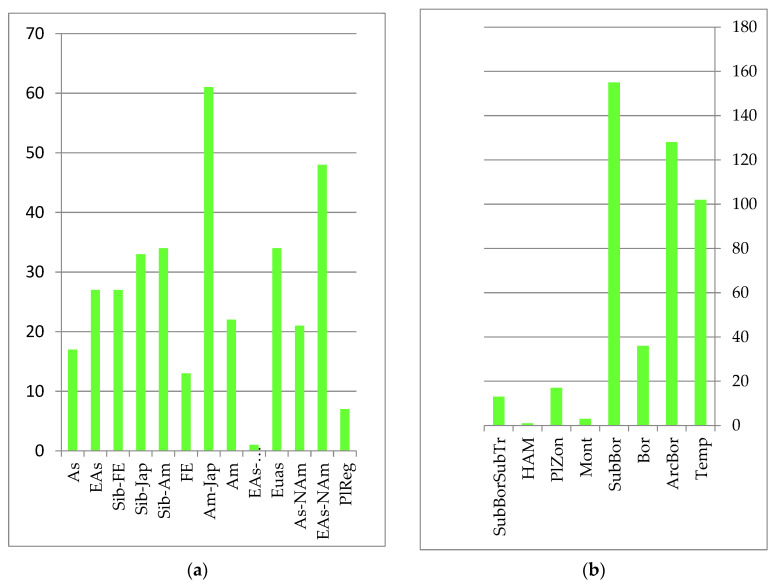
Number of species groups of the meadow floristics complex: (**a**) longitudinal species groups: Asian (As); Eastern Asian (EAs); Siberian–Far Eastern (Sib-FE); Siberian–Japanese (Sib-Jap); Siberian–Amurense (Sib-Am); Far Eastern (FE); Amurense–Japanese (Am-Jap); Amurense (Am); Eastern Asian–Paleotropical (EAs-PalTrp); Eurasian (Eas); Asian–North American (EAs-Nam); Eurasian–North American (Eas-NAm); PlReg–pluriregional; (**b**) zonal species groups: temperate (Temp); arcto–boreal (ArcBor); boreal (Bor); sub-boreal (SubBor); montane (Mont); plurizonal (PlZon); hypoarcto–montane (HAM); sub-boreal–sub-tropical (SubBorSubTr).

Floodplain reed-sedge and reed meadows are of landscape-forming importance. The main coenose-formers in floodplain meadows are *Calamagrostis langsdorffii* (Link) Trin. (*Calamagrostis purpurea* (Trin.) Trin.) and *C. angustifolia* Kom, which form predominantly monodominant communities. Less widely distributed steppe legume–forb–grass and upland wormwood–couch grass, cereal–forb and forb meadows are located mostly on the secondary, high alluvial floodplains and flat rivers of the Amur River floodplain. The autochthonous core is formed from species with Amur, Amur–Japanese, Siberian–Japanese and Siberian–Amur ranges of the sub-boreal belt-zonal group (see Figure 3): *Cynoctonum purpureum* (Pall.) Pobed. (*Cynanchum purpureum* (Pall.) K. Schum.), *Ixeridium gramineum* (Fisch.) Tzvel. (*Ixeris chinensis* subsp. *versicolor* (Fisch. ex Link) Kitam), *Lilium callosum* Siebold et Zucc., *Arundinella anomala* Steud. (*Arundinella hirta* (Thunb.) Tanaka, and *Hemarthria sibirica* (Gand.) Ohwi.

Specific halophilic shortgrass meadows are common in the Amur Estuary, on the coast of the Tatar Strait and Sakhalin Bay. These meadows form a group of halophilic species, including *Puccinellia phryganodes* (Trin.) Scribn. et Merr., *Triglochin asiatica* (Kitag.) Á. Löve et D. Löve (*Triglochin maritima* L.), *Poa macrocalyx* Trautv. et C.A. Mey., and *Stellaria humifusa* Rottb.

#### 2.2.3. Mire Floristic Complex

The mire floristic complex comprises 74 species. The spectrum of leading families is dominated by *Cyperaceae* (34 species), *Cyperaceae* (34), *Ericaceae* (9), *Eriocaulaceae* (4), *Droseraceae, Orchidaceae*, *Salicaceae* (3), *Betulaceae*, *Rosaceae* and *Scrophulariaceae* (2).

**Figure 4:**
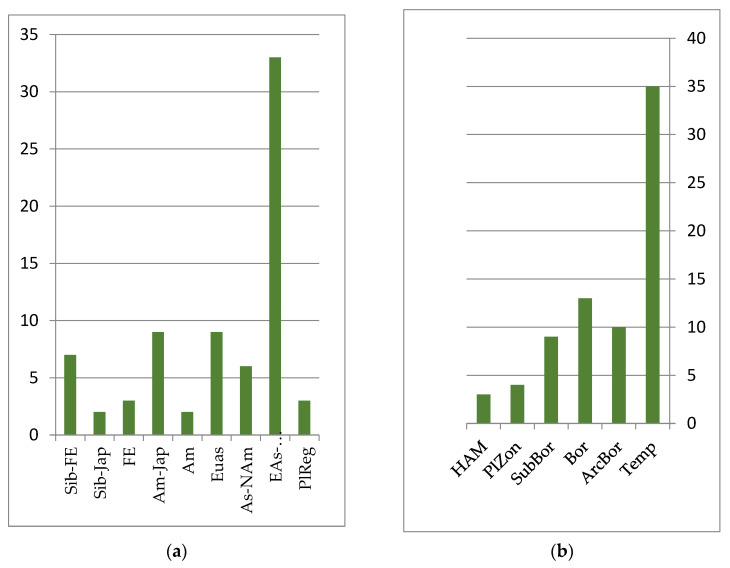
Number of species groups of the mire floristics complex: (**a**) longitudinal species groups: Siberian–Far Eastern (Sib-FE); Siberian–Japanese (Sib-Jap); Far Eastern (FE); Amurense–Japanese (Am-Jap); Amurense (Am); Eurasian (Eas); Asian–North American (EAs-Nam); Eurasian–North American (Eas-NAm); PlReg–pluriregional; (**b**) zonal species groups: temperate (Temp); arcto–boreal (ArcBor); boreal (Bor); sub-boreal (SubBor); plurizonal (PlZon); hypoarcto–montane (HAM).

The mire flora is monotonous and not species-rich throughout the Lower Amur region. In the ecological–coenotic groups of heterotrophic, mesotrophic sphagnum and tree–sphagnum bogs, 73 species were noted, while in oligotrophic, eutrophic grass and tree–sphagnum bogs, 71 were seen. Representatives of the swamp floristic complex are characterized by significant Eurasian and Eurasian–North American pluriregional ranges, covering mainly the northern part of the temperate zone of Eurasia and North America (see Figure 4). These include species such as *Ledum palustre* L. (*Rhododendron tomentosum* Harmaja), *Andromeda polifolia* L., *Carex lapponica* O. Lang, *Drosera rotundifolia* L., etc.

#### 2.2.4. Mountain Tundra Floristic Complex

The high-altitude position in the mountain systems of the Lower Amur region is occupied by representatives of the high mountain, or the mountain–tundra floristic complex. The species diversity of plants in this complex comprises 223 taxa (12.5% of the flora), among which representatives of the families *Cyperaceae* (27 species), *Asteraceae* (25), *Poaceae* (19), *Saxifragaceae* (17), *Salicaceae* (14), *Ericaceae* (13), *Caryophyllaceae, Rosaceae* (12), *Polygonaceae* (10), and *Scrophulariaceae* (9) predominate in terms of the number of species.

**Figure 5:**
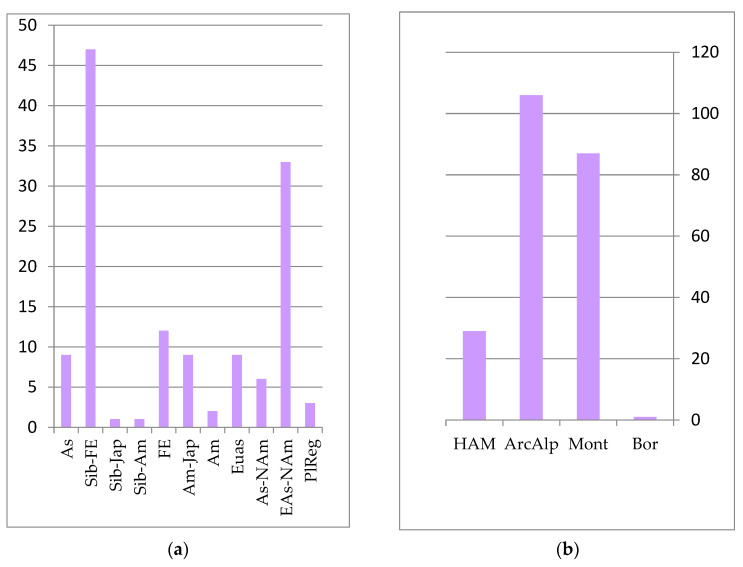
Number of species groups of the mountain tundra floristics complex: (**a**) longitudinal species groups: Asian (As); Siberian–Far Eastern (Sib-FE); Siberian–Japanese (Sib-Jap); Siberian–Amurense (Sib-Am); Far Eastern (FE); Amurense–Japanese (Am-Jap); Amurense (Am); Eurasian (Eas); Asian–North American (EAs-Nam); Eurasian–North American (Eas-NAm); PlReg–pluriregional; (**b**) zonal species groups: boreal (Bor); montane (Mont); arcto–alpine (ArcAlp); hypoarcto–montane (HAM).

The intrazonal nature of the mountain–tundra complex is emphasized by the vast longitudinal ranges (Eurasian, Eurasian–North American, Asian–North American, Siberian–Far Eastern, etc.) of most of their representatives, which belong to the belt-zonal arcto-alpine, arcto-boreal and hypoarcto-montane groups of species, whose ecological optima are located at higher latitudes (see Figure 5). Within the boreal and sub-boreal zones, these taxa are distributed over mountain systems. The vegetation in the upper levels of the mountains of the Lower Amur region belongs to the tundra, shrub, forest, marsh, and meadow types [23,42].

#### 2.2.5. Rocky-Scree Floristic Complex

The species diversity of plants in scree habitats is dominated by the families *Poaceae* (18 species), *Caryophyllaceae* (18), *Lamiaceae*, *Brassicaceae*, *Asteraceae* (14), *Saxifragaceae* (9), *Selaginellaceae* (7), *Rosaceae* (6), *Woodsiaceae* and *Crassulaceae* (4). The flora of the rocky-scree floristic complex comprises 507 species, of which 158 are obligate petrophiles, and the others are facultative, representatives of forest, meadow and mountain–tundra floristic complexes.

**Figure 6:**
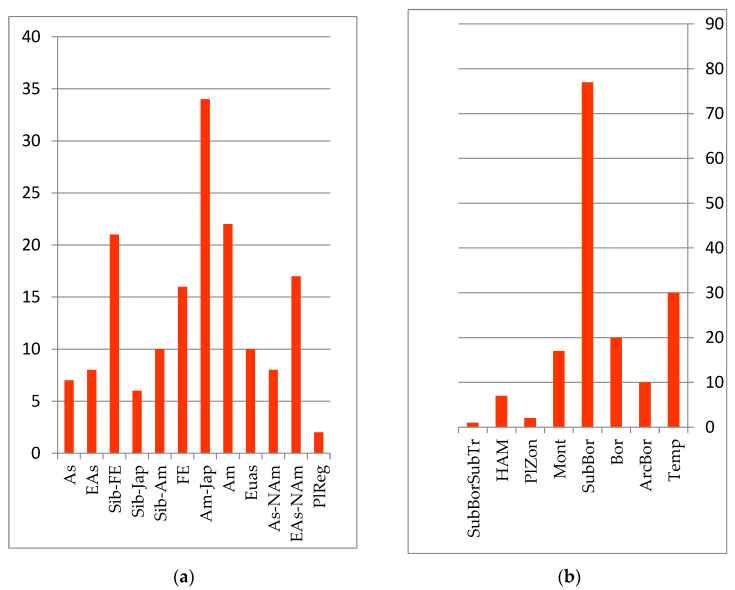
Number of species groups of the rocky-scree floristics complex: (**a**) longitudinal species groups: Asian (As); Eastern Asian (EAs); Siberian–Far Eastern (Sib-FE); Siberian–Japanese (Sib-Jap); Siberian–Amurense (Sib-Am); Far Eastern (FE); Amurense–Japanese (Am-Jap); Amurense (Am); Eurasian (Eas); Asian–North American (EAs-Nam); Eurasian–North American (Eas-NAm); PlReg–pluriregional; (**b**) zonal species groups: temperate (Temp); arcto–boreal (ArcBor); boreal (Bor); sub-boreal (SubBor); montane (Mont); plurizonal (PlZon); hypoarcto–montane (HAM); sub-boreal–sub-tropical (SubBorSubTr).

Rocky, flinty habitats are demanding for plants, and are determined by poor skeletal soils that form locally, strong solar radiation, high temperatures of substrates, an air layer, etc. This group of plants is quite specific. Sub-boreal species predominate in Amur, Amur–Japanese and Siberian–Far Eastern areas (see Figure 6). The main coenose-formers are xerophilic representatives of *Thymus*, *Clematis*, *Agastache*, *Rabdosia*, *Vincetoxicum* and *Koeleria cristata* (L.) Bertol. (*Rostraria cristata* (L.) Tzvelev), *Melica turczaninowiana* Ohwi, *Poa ochotensis* Trin. (*Poa versicolor* Besser), etc.

#### 2.2.6. Aquatic–Semiaquatic Floristic Complex

**Figure 7:**
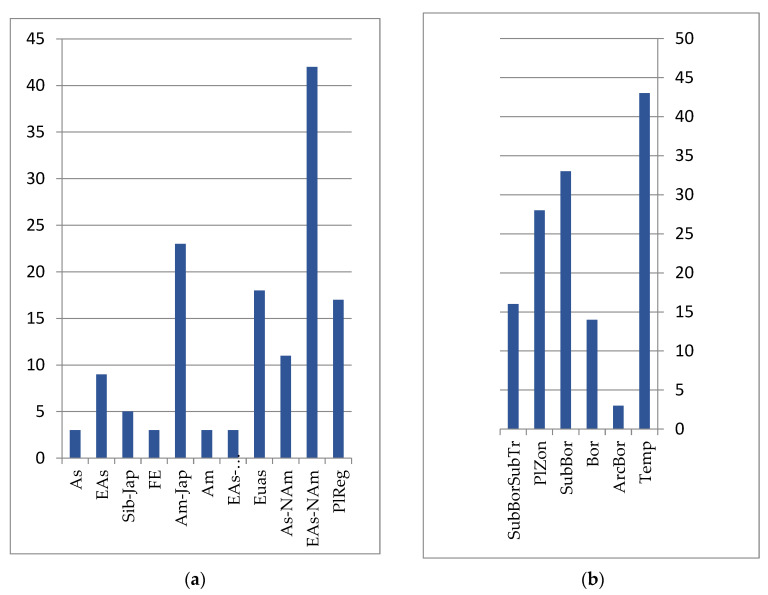
Number of species groups of the aquatic–semiaquatic floristics complex: (**a**) longitudinal species groups: Asian (As); Eastern Asian (EAs); Siberian–Far Eastern (Sib-FE); Far Eastern (FE); Amurense–Japanese (Am-Jap); Amurense (Am); Eastern Asian–Paleotropical (EAs-PalTrp); Eurasian (Eas); Asian–North American (EAs-Nam); Eurasian–North American (Eas-NAm); PlReg–pluriregional; (**b**) zonal species groups: temperate (Temp); arcto–boreal (ArcBor); boreal (Bor); sub-boreal (SubBor); plurizonal (PlZon); sub-boreal–sub-tropical (SubBorSubTrp).

The floristic complex of aquatic–semiaquatic species includes 137 taxa, among which are representatives of the families *Cyperaceae* (18 species), *Potamogetonaceae* (15), *Trapaceae, Sparganiaceae* (7), *Lemnaceae*, *Poaceae*, *Polygonaceae*, *Ranunculaceae* (6), *Alismataceae*, *Haloragaceae*, *Najadaceae*, *Nymphaeaceae*, *Zosteraceae*, *Asteraceae*, *Typhaceae* and *Lentibulariaceae* (4). The specificity of the water–coastal complex contains the groups of Amur–Japanese, Amur and Siberian–Amur species of the sub-boreal belt–zonal area: *Caulinia japonica* (Nakai) Nakai (*Najas gracillima* (A. Braun ex Engelm.) Magnus), *Trapa japonica* Fler. (*Trapa natans* var. *bispinosa* (Roxb.) Makino), and *Trapella sinensis* Oliv. (see Figure 7).

#### 2.2.7. Floodplain–Estuarine Shallow Floristic Complex

**Figure 8:**
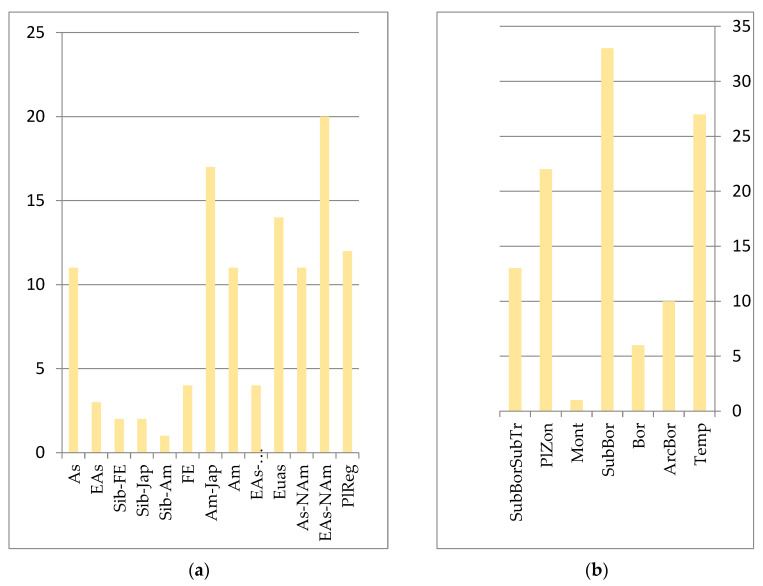
Number of species groups of the floodplain–estuarine shallow floristics complex: (**a**) longitudinal species groups: Asian (As); Eastern Asian (EAs); Siberian–Far Eastern (Sib-FE); Siberian–Japanese (Sib-Jap); Siberian–Amurense (Sib-Am); Far Eastern (FE); Amurense–Japanese (Am-Jap); Amurense (Am); Eastern Asian–Paleotropical (EAs-PalTrp); Eurasian (Eas); Asian–North American (EAs-Nam); Eurasian–North American (Eas-NAm); PlReg–pluriregional; (**b**) zonal species groups: temperate (Temp); arcto–boreal (ArcBor); boreal (Bor); sub-boreal (SubBor); montane (Mont); plurizonal (PlZon); sub-boreal–sub-tropical (SubBorSubTrp).

The floristic complex of the floodplain–estuarine shallow species growing in the floodplain of the Amur River, and in estuarine areas of the sea coast, is scarce. It comprises 112 taxa. The families *Poaceae* (17 species), *Cyperaceae* (16), *Polygonaceae* (13), *Astreaceae* (10), *Chenopodiaceae*, *Brassicaceae* (7), *Scrophulariaceae* and *Juncaceae* (6) are the leading species in terms of diversity. The independence of this complex is determined by its diversity, and the narrowness and specificity of the growing conditions. The Far Eastern nature of the hydrological regimes of rivers, with a weak spring flood and a well-pronounced flood regime with a long period of low water, determine the formation of specific ecotopes of the river shoals during this period; conditions to which part of the flora of the Lower Amur region is adapted to grow. This is represented by *Rorripa globosa* (Turcz. ex Fisch. et C.A. Mey.) Hayek, *Scutellaria strigillosa* Hemsl., *Rumex amurensis* Fr. Schmidt ex Maxim. and *Corispermum elongatum* Bunge ex Maxim. Their originality is characterized by the dominance of species with Amur, Amur–Japanese and Siberian–Japanese ranges in the sub-boreal belt–zonal element (see Figure 8).

Under the conditions of soil salinization as a result of seawater penetrating from the Amur Estuary via tidal processes and surf action, somewhat different communities develop in the supralittoral zone and in the shallows, where halophytic species occupy leading positions. Their diversity is insignificant, represented by *Ligusticum scothicum* L., *Chorisis repens* (L.) DC., *Mertensia maritima* (L.) Gray, *Atriplex subcordata* Kitag., *Arctopoa eminens* (J.S. Presl) Prob., *Polygonum boreale* (Lange) Small and *Glaux maritima* L. (*Lysimachia maritima* (L.) Galasso, Banfi et Soldano) forming open communities on the shallow and variably waterlogged shores.

### 2.3. Regularities of the Plant Covers’ Spatial Structure

The regularities of some floristic complexes are defined by the landscape’s ecological conditions, and the variety in the edaphic, orographic and climate parameters within the region. The floristic complexes’ allocation within the composition of the vegetation in the altitudinal and latitudinal ranges of the Lower Amur region is associated with differences in climatic conditions (heat supply during the vegetation period, the ratio of heat and moisture, the accumulation of average daily temperatures above 10 °C), the lithology (groups of rocks, their formation conditions, the geochemical flow processes), the position in the relief (the altitude above sea level, northern or southern slope exposure, steepness, valley form, etc.), hydrological factors (the stability and variability of the water regime, the groundwater-level, the soil water–air regime, etc.), along with seed and material exchanges in ecosystems.

**Figure 9:**
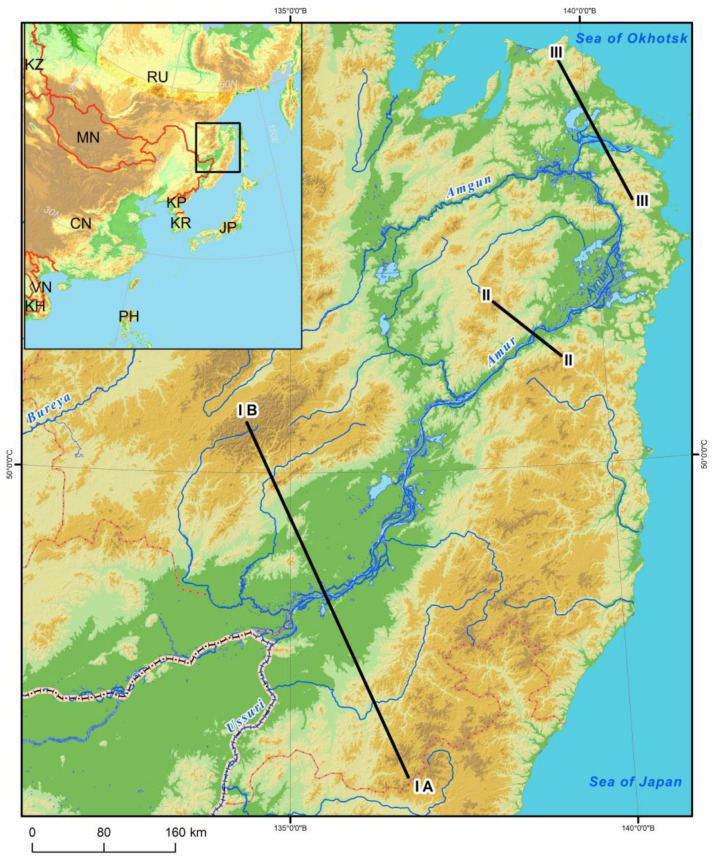
Map showing the position of hypsometric profiles within the Lower Amur region.

The Lower Amur River Valley ranges across flat, low-mountain and mid-mountain territories differing in hydrological regimes, climate and vegetation. For the plant cover structural analysis, we considered three profiles crossing the Amur River Valley in the mountain sections of Ko–Ulun, Pik–Brandibrat and My–Orel (see Figure 9).

**Figure 10:**
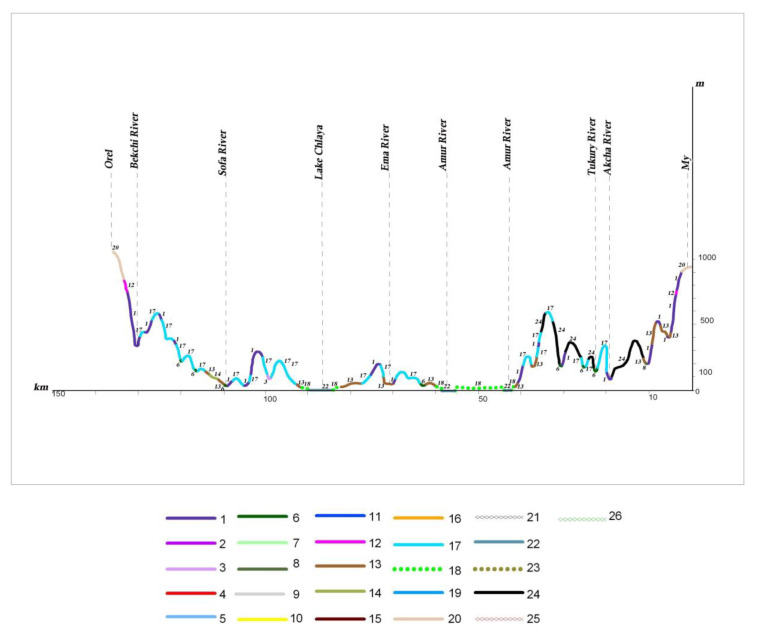
Profiles of plant cover from the mountain My to the mountain Orel (III): 1—spruce and fir–spruce green moss, small herb–green moss mountain forests and subalpine sparse forests in some places combined with stone birch forests; 2—spruce and fir–spruce green moss, small herb–green moss, green moss–fern mountain forests; 3—fir–spruce–green moss–fern and herb–fern with nemoral elements in the cover and undergrowth of the slopes of mountain and submontane forests in some places, with *Pinus koraiensis* and broadleaf species; 4—Korean pine–broadleaf shrub–mixed herb, shrub–mixed herb–fern, liana–mixed herb–fern mountain and submontane forests, in some places combined with coniferous species; 5—mixed (polydominant) broadleaf mountain and submontane secondary forests in situ of the Korean pine broadleaf forests; 6—the range of willow stand–chosenia–poplar floodplain vegetation in some places combined with broadleaf elm–ash valley forests; 7—the range of willow stand–chosenia–poplar floodplain vegetation in some places combined with fir–spruce green moss, herb and herb–shrub valley forests; 8—the range of willow stand–alder–poplar floodplain vegetation in some places combined with shrub thickets and meadows; 9—oak submontane, mountain forests with participation by other broadleaf species; 10—oak forests with participation by other broadleaf species, sparse shrub thickets on the alluvial floodplain terraces, eolian ridges; 11—stone birch herb subalpine forests; 12—creeping dwarf pine and dwarf alder subalpine forests, in some places combined with mountain tundra; 13—larch ledum, herb–dwarf shrub mountain forests; 14—fen, swamp, bogs combined with larch shrubby birch–sphagnum, oligotrophic dwarf shrub, shrub–sphagnum, sedge–sphagnum waterlogged forests and sparse forests, shrubby birch and willow stand waterlogged thickets on the floodplain and marine terraces; 15—larch green moss–ledum, herb valley forests and sparse forests; 16—larch mesophyllus herb–shrub forests with participation by the nemoral elements in the herb cover and slope in some places combined with oak and black birch; 17—white birch herb secondary valley and submontane forests in situ with larch and fir–spruce forests, in some places combined with shrubby birch thickets and meadows; 18—white birch and aspen herb, herb–shrub secondary mountain, submontane forests in situ Korean pine broadleaf, fir–spruce forests in some places combined with mixed broadleaf, oak–white birch forests, sparse forests and shrub thickets; 19—sedge–reedgrass, reedgrass, herb–grass periodically and constantly wet meadows in some places combined with meadow–shrub communities; 20—cryophilic rocky, lichen, polygonal, spotty, dwarf shrub–lichen and dwarf shrub mountain tundra in some places combined with dwarf pine and dwarf alder thickets, cryophilic alpine grassland; 21—grass, sagebrush, grass–herb communities; 22—herbal communities of the floating, submerged, coastal aquatic and subaquatic type; 23—agricultural land in some places with valley and floodplain meadows; 24—burning; 25—clearcut forest areas; 26—post-pyrogenic herb–shrub communities.

On the plains within the southern taiga (profile III) (see Figure 10), sparse sphagnum, sedge–sphagnum fen with larch (*Larix cajanderi*), and birch (*Betula platyphylla* Sukacz. (*Betula pendula* subsp. *mandshurica* (Regel) Ashburner et McAll.) forests are common in low and waterlogged areas, which are periodically flooded. The sphagnum, shrub–sphagnum swamps and shrub–sphagnum waterlogged larch sparse forests, with *Betula fruticosa* Pall., *Vaccinium uliginosum* L., *Salix brachypoda* (Trautv. et C.A. Mey.) Kom., *Chamaedaphne calyculata* (L.) Moenchand and *Pinus pumila* (Pall.) Regel in the shrub and herb–dwarf shrub layers, develop in some waterlogged places close to areas of permafrost but that are less affected by flooding. They alternate with sedge fen.

**Figure 11:**
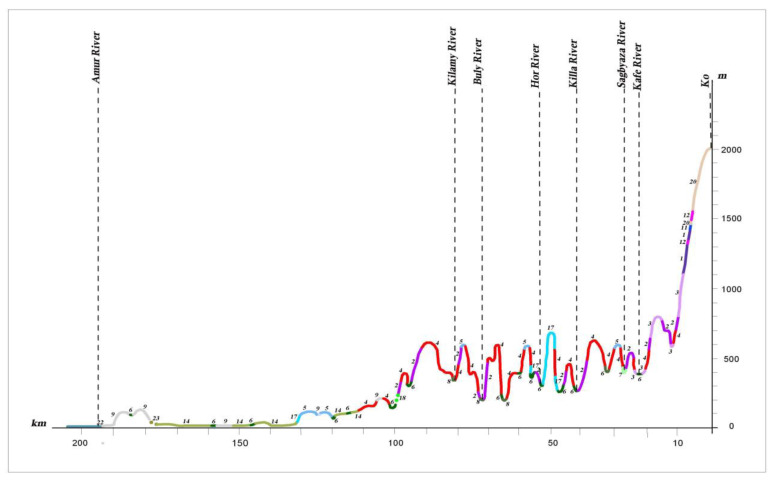
Profile of the plant cover from the mountain Ko to the Amur River (I A) (see the legend in the Figure 10).

**Figure 12:**
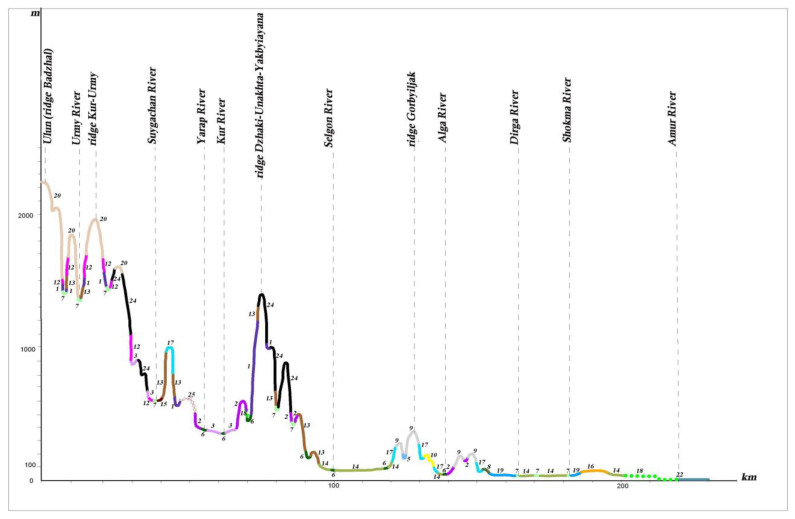
Profile of the plant cover from the Amur River to the mountain Ulun (I B) (see the legend in the Figure 10).

The larch herb forests and aulocomnium–sphagnum larch sparse forests with degraded specimens of *Picea ajanensis* can be found on waterlogged plain territories within the coniferous–broadleaf forest zone (profile I A, B) (see Figure 11 and Figure 12). The waterlogged larch are more commonly found with species *Salix*, *Alnus* and *Betula* under their canopy on poorly drained watersheds, gentle slopes and high floodplains. As the swamping process increases, these subtaiga larch forests are replaced by willow and birch thickets and herb–moss fen. Most of the floodplain territories of the Middle Amur lowland are occupied by meadows–shrub thickets–forest community ranges (profiles I A and B). Under these conditions, relief and flood regimes have a great influence on vegetation distribution.

In wide valleys with gentle slopes, the change from waterlogged larch, small-leaf forests (white birch, aspen) and meadow–swamp communities on the river valley bottom to typical taiga vegetation is gradual.

In the more favorable climate and edaphic conditions of the low remnant mountains, the mountain foothills, and the lower parts of mountain slopes, Korean pine–broadleaf forests are found (profile I A, B). Near the northern boundaries of distribution, within the 50°–51°30′ northern latitude, they occur only in small scattered patches. In the Lower Amur River basin, Korean pine–broadleaf forests are represented mainly by their northern variants—Korean pine–broadleaf forests with significant additions of *Picea ajanensis* and *Abies nephrolepis*. 

To the north, Korean pine–broadleaf and coniferous–broadleaf forests concede their habitat to subtaiga larch, fir–spruce and white birch forests. Oak and larch–oak forests with *Pinus pumila* under the canopy are common on mountain slopes only along the Amur River Valley up to its estuary.

**Figure 13:**
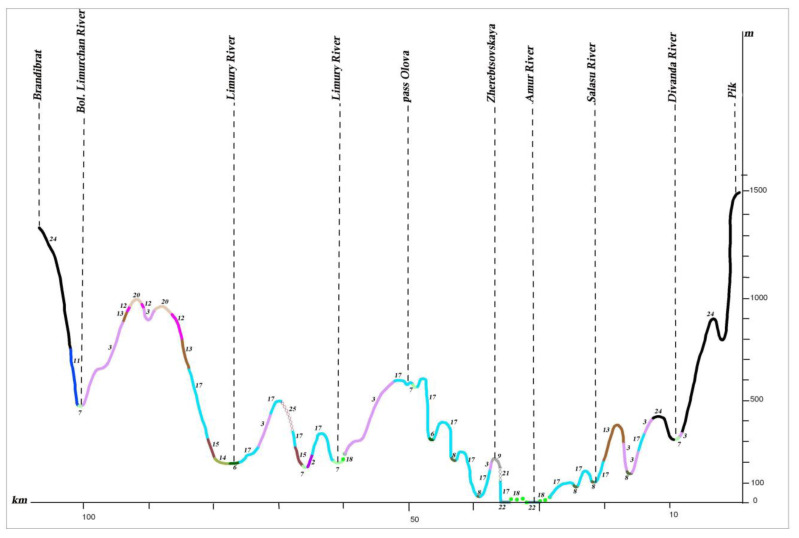
Profile of the plant cover from the mountain Pik to the mountain Brandibrat (II) (see the legend in the Figure 10).

Dark coniferous green moss, small herb and small herb–dwarf shrub forests are common on the mountain slopes of different exposures. They are spread on the low-mountain, low-hill and ridged reliefs of volcanic plateaus up to the 1000–1500 m altitudinal ranges, and are most typical of the northern part of the Lower Amur region (profiles II, III) (see Figure 10 and Figure 13). Compared to fir–spruce green moss forests, fir–spruce forests with nemoral elements are much less common. These forests achieve the greatest development in Sikhote–Alin (profile I A) (see Figure 11). They extend in a narrow strip along the Amur River Valley to the north to the Limuri River Estuary, and can be noted in the Bolchoye Kizi Lake basin [27,28].

On the mountain slopes of the Bureya Highlands ridge (Badzhal, Dusse–Alin and Yam–Alin), larch–spruce and larch forests are characterized by wide distribution, and on intermountain depressions and wide mountain valleys on cold and partially waterlogged bottom and slopes, larch moss–dwarf shrubs and ledum forests dominate. They spread into plant cover on these mountain systems, as noted by many authors [26,27,49], marking the boundary between dark coniferous taiga and light coniferous–larch taiga.

Dark coniferous forests on high mountains change into subalpine sparse forests, which are formed from *Picea ajanensis*, *Larix cajanderi* and *Betula lanata* (Regel) V. Vassil. (*Betula ermanii* var. *lanata* Regel) with undergrowth comprising *Pinus pumila*, *Weigla middendorffiana* (Carrière) K. Koch and *Rhododendron aureum* Georgi. They meet in a narrow strip near the upper forest limit of the highest peak or watershed of the Sikhote–Alin mountain system, and on ridges between the Amur River and Amgun River, and the Amgun River and Okhotsk Sea (profile I A) (see Figure 11). On the ridges of Badzhal, Dusse–Alin and Yam–Alin, the 2000 m altitudinal range (profile I B) (see Figure 12) of subalpine sparse forests is more developed, and the proportion of *Larix cajanderi* also increases. Upper subalpine sparse forests change to dwarf forests of *Pinus pumila*, which are spread across the peaks under the conditions of the low mountains of the Bureya Highlands (profile I B, II, III) (see Figure 10, Figure 12 and Figure 13). The dwarf forests of *Pinus pumila* form in the altitudinal belt from the 1400–1700 m to the 1900–2000 m altitudinal range on the ridges Badzhal, Dusse–Alin and Yam–Alin. Mountain tundra is common on the highest mountains, where tundra is characterized as distributed intermittently. This is due to differences in elevation, separated by saddles.

The different types of the mire and waterlogged sparse forest communities are characterized by a significant distribution on the waterlogged plains in the northern part of the Amur River basin, in the Amgun River basin, and on the coast near the Amur River Estuary. *Pinus pumila*, *Empetrum sibiricum* V. Vassil. (*Empetrum nigrum* subsp. *stenopetalum* (V.N. Vassil.) Nedol.), *Carex cryptocarpa* C.A. Mey. (*Carex lyngbyei* Hornem.), *C. chordorrhiza* L. f., *C. rariflora*, *Salix fuscescens* and *Pedicularis lapponica* L. (Figure 10) are present in the composition.

In recent decades, the structure of the natural plant cover has been significantly transformed by human activity. The effect of anthropogenic change on vegetation complexes increased after the 1920s–1930s, due to the intensive development of the Amur River basin. Logging, the reclamation of land suitable for agriculture, the development of mining and processing enterprises, the construction of linear structures (roads, gas and oil pipelines, power lines), and the associated increase in the pyrogenic factor led to more than 70% of the territory now being represented by variously transformed derivative vegetation groups, which have replaced the natural zonal forest, meadow, mire, and other communities. In the conditions of the middle and southern taiga, they change to larch and often waterlogged forests (profiles II, III) (see Figure 10 and Figure 13). In the southern part of the territory, the role of the light coniferous forests has decreased, and small-leaf forests appear in place of the Korean pine–broadleaf, coniferous–broadleaf and dark coniferous forests. These are formed from *Betula platyphylla* and *Populus tremula*, and the broadleaf species *Quercus mongolica*, *Betula costata*, *B. davurica* and *Ulmus macrocarpa* Hance.

## 3. Discussion

The vascular plant flora of the Lower Amur region includes 2240 species, comprising 760 genera and 158 families. The native flora includes 1801 species, comprising 602 genera and 152 families. The group of adventive plants is represented by 439 species, comprising 256 genera and 56 families, which comprise 19.6% of all flora. The flora is defined by the combination of the parameters of the boreal and sub-boreal East Asian floras.

The range of leading families characterizes the Lower Amur region flora as arctic–boreal–East Asian, with meadow–mountain features. Forest humid areas are defined by the high species diversity of the *Lamiaceae*, *Scrophulariaceae*, *Orchidaceae*, *Apiaceae*, *Violaceae*, *Campanulaceae*, *Convallariaceae*, *Liliaceae* and *Rubiaceae* families. The uniqueness of the Lower Amur region’s flora is clear at the genera and species levels, and is defined by a combination of the elements of the boreal and sub-boreal floras.

In the Lower Amur region, the species of the forest floristic complex predominate, which is explained by their full compliance with present climate conditions. This region is an arena for the complex interactions between the different geneses of the Okhotsko–Kamchatense, Angaridense, Amurense, Beringian and Siberian forest formations.

The core of the floristic complexes of the Lower Amur region is formed by species whose formation and distribution arez associated with East Asia. They are representatives of the Eastern Asian (6.3% of the total number of native flora), Far Eastern (6.3%), Amurense–Japanese (18.2%) and Amurense longitudinal species groups (10%). 

Florogenetic relationships amongst Asian, European and North American floras are highlighted by species with extensive areas—pluriregional (2.6% of the total number of native flora), Eurasian–North American (16.7%), Asian–North American (7.1%), Eurasian (9.4%), Eastern Asian–Paleotropical (0.5%), Asian (3.8%), Siberian–Japanese (5.3%), Siberian–Far Eastern (10.1%) and Siberian–Amurense longitudinal species groups (3.7%). These species are edificators in dark and light coniferous zonal forests, intrazonal and azonal mires, meadows, and rocky scree aquatic–semiaquatic floristic complexes: *Larix cajanderi*, *Picea obovata*, *Populus tremula*, *Calamagrostis langsdorffii*, *Ledum palustre*, *Betula fruticosa*, etc.

The forest, meadow, and rocky-scree floristic complexes are characterized by the greatest originality. In total, 34.9%, 31.2% and 37.9% of the species diversity of these complexes are represented by Eastern Asian, Far Eastern, Amurense–Japanese and Amurense longitudinal species groups. The species in these groups occupy the dominant position, and often function as edificators in vegetation communities: *Pinus koraiensis*, *Abies nephrolepis*, *Picea ajanensis*, *Tilia amurensis*, *Fraxinus mandshurica*, *Betula costata*, *Quercus mongolica*, *Acer mono*, *Miscanthus sacchariflorus* (Maxim.) Benth. et Hook. f. ex Franch., *Glyceria leptorhiza* (Maxim.) Kom., *Kalimeris incisa* (Fisch.) DC. (*Aster incisus* Fisch.), *Zizania latifolia* (Griseb.) Hance ex F. Muell., *Aster tataricus* L. f., *Poa ochotensis*, *Thymus nervulosus* Klokov, etc. Some of these species are relics; under modern conditions, they are threatened and occur in small populations: *Panax ginseng*, *Ilex rugosa* F. Schmidt, *Phyllitis japonica*, *Coniogramme intermedia* Hieron., *Macrohystrix komarovii* (Roshev.) Tzvel. et Probat. (*Leymus komarovii* (Roshev.) J.L. Yang et C. Yen), *Pleopeltis ussuriensis* Regel et Maack (*Lepisorus ussuriensis* (Regel et Maack) Ching), *Taxus cuspidata*, etc.

The florogenetic relationships in the subarctic high mountains of Asia and North America are mostly represented by the tundra–mountain complex (56% and 22% of species diversity of this complex, respectively), and the relationships on the enormous northern waterlogged plains are represented by the mire floristic complex (22.5% and 20.3% species diversity in this complex, respectively). These are relict elements of the cold periods of the Pleistocene, and are evident in the ecotone “taiga–coniferous–broadleaf forests” in the Amur River basin.

The location of the territory within the zonal ecotone “taiga–coniferous–broadleaf forests” is reflected in the diversity of zonal elements. The ratios of temperate, arcto-boreal and boreal elements and sub-boreal elements in the forest complex composition are almost proportional (40.2% and 49.3% of species diversity in this complex, accordingly). The temperate, arcto-boreal and boreal species zonal groups occupy important positions in the meadow and mire forest complexes (77.1% and 78.4% of the species diversity of these complexes, accordingly).

In addition to the zonal environmental factors determined by the geographical position of the region, azonal factors and processes are at play. These include floodplain, alluvial, karst and other processes that determine the formation of specific groups of rocky-scree, floodplain-estuarine shallow, and aquatic–semiaquatic floristic complexes in the Lower Amur.

Our studies of the spatial structure of the vegetation cover’s diversity in the Lower Amur region show that the distribution of vegetation depends on many factors but, above all, on the climate.

The warming effect of water flows, and the meridional position of the mountain ranges, perpendicular to the monsoonal movement of air masses that come during the vegetation period from the tropical regions of the Pacific Ocean, leads to a shift in the vegetation of the corresponding zones and subzones in the Amur River Valley. As such, nemoral coniferous–broadleaf forests intrude into the south taiga. 

The maximum rates of occurrence and abundance are noted for sub-boreal thermophilic East Asian elements of the flora in the southern part of the region, at 134–137° E, and at 50–51° N within the low-mountain zones of the coniferous–broadleaf and broadleaf forests. These contain the highest number of indicators of species diversity—more than 800 species. The core relic species are concentrated in the formations of the northern types of the coniferous–broadleaf forests and nemoral fir–spruce forests: *Deutzia amurensis* (Regel) Airy Shaw (*Deutzia parviflora* var. *parviflora* Bunge), *Ilex rugosa*, *Aralia elata*, *Plagiorhegma dubium* Maxim., *Arisaema amurense*, *Chloranthus japonicus* Siebold (*Chloranthus serratus* (Thunb.) Roem. et Schult.), *Phyllitis japonica*, *Caragana ussuriensis* (Regel) Pojark., etc. Most of these species are distributed in the southern regions. An insignificant part of the sub-boreal flora has adapted to the suboceanic sector of the Lower Amur region: *Taxus cuspidata*, *Schisandra chinensis* (Turcz.) Baill., *Gastrodia elata* Blume, *Gagea nakaiana* Kitag., *Ligustrina amurensis* Rupr. (*Syringa reticulata* subsp. *amurensis* (Rupr.) P.S. Green et M.C. Chang), *Vitis amurensis* Rupr., etc.

Azonal forest–steppe elements, thermophilic elements, and aquatic, mire and meadow floristic complexes also permeate into the northeast along the Amur River Valley: *Bupleurum scorzonerifolium* Willd., *Leontopodium conglobatum* (Turcz.) Hand.-Mazz., *Vincetoxicum atratum* (Bunge) C. Morren et Decne., *Filifolium sibiricum* (L.) Kitam., *Rhynchospora fujiiana* Makino, *Eriocaulon chinorossicum* Kom., *Hemerocallis coreana* Nakai, *Ottelia alismoides* (L.) Pers., *Nelumbo nucifera* Gaertn., *Euryale ferox* Salisb., etc.

The formation of the vegetation cover of the northeastern and eastern parts of the Lower Amur River basin was influenced by the severity of the Okhotsk Sea. A decrease in species diversity is observed in the boreal dark and light coniferous forests, with the replacement of coniferous–broadleaf forests on mountain slopes and in river valleys, and in the compositions of meadow and mire communities.

The diversity and structure of the vegetation cover of the Low Amur region in the last century have been significantly affected by economic activity and fires. Together, these factors have contributed to almost 70% of the territory being represented by variously transformed derivative vegetation groups that have replaced the natural zonal forest, meadow, mire, and other communities. The processes of the ecotonization of the vegetation cover continue to intensify, including areal fragmentation processes, reductions in the number, occurrence and vitality of natural species populations, and the emergence of groups of synanthropic and invasive species.

## 4. Materials and Methods

The plant cover of the Lower Amur region is the object of study, and the regularity of the species diversity structure and the plant cover in the zonal–climate ecotone “taiga–coniferous–broadleaf forests” and the boreal–nemoral ecotone is the research subject.

The study of the plant cover considers the ecological and geographical structure of the ecotone plant cover, which involves the use of floristry, geography and botany methods. To study the plant cover, field descriptions were made from 1993 to 2021. Surveys were conducted annually in the growing season from May to September. About 90 expeditions were organized to various parts of the Lower Amur region. The methodological basis of the research consists of the traditional techniques of floristic and geobotanical study [50,51,52].

In our results, we collected over 15,000 herbarium samples and 1400 floristic and geobotanical descriptions were carried out on the different vegetation plots.

For this purpose, based on data from the literature, maps and satellite images, the selection of individual plots was made at the preparation stage, taking into account the full coverage of geomorphological sections of the territory. The individual plots were selected from an end-to-end transection of the Amur River Valley, from south to north, and through the river valley from west to east, so as to cover the various communities on the different landforms. Ordination, along latitudinal, longitudinal and altitudinal gradients of physical, geographical, and climatic factors, was performed. This made it possible to identify patterns of spatial changes in the species diversity of various ecotopes under the conditions of ecotonal territories [5,53].

The author identified the species richness of communities (individual plots ranging from 100 to 1000 m^2^, determined by the area of the community required to identify species richness) and its activity (an integral indicator of the occurrence and abundance of vascular plant species). The separation of the core of the floristic complexes or, in other words, species demonstrating the highest fidelity to different floristic complexes was conducted by applying the Braun-Blanquet scale [54]. The herbarium collection has been preserved in the Herbarium of the Institute of the Water and Ecology Problems Far-East Branch of the Russian Academy of Science (KHA).

A list of flora was compiled from the results of field research and the treatment of the herbarium collection, and this was published in 2013 [11]. The names of the species studied are mainly assigned according to the vascular plant lists of the Far East [13,14,15,16,17,18,19,20,21]. The plant names of the species according to Plants of the World Online are given in parentheses whenever they are mentioned in the text for the first time if they are different.

All descriptions were input into an electronic database with MS Excel software.

For the analyses of flora, we used the traditional methods of floristic research [40,46,55]. The diversity and structure of the flora (taxonomic, geographic, altitudinal–belt–biological and ecological–coenotic elements), the features of its spatial distribution, and its current state were studied.

## Figures and Tables

**Table 1:** Structure of the head family spectrum according to percentage of plant species in a family.

Vascular Plant Flora	Family Rank									
	1	2	3	4	5	6	7	8	9	10
								Rank 8–9		
Total fora	As	Poa	Cy	Ro	Ra	Br	Pol	Fa	Ca	La
	12.7	8.9	7.7	4.3	4.1	3.4	3.4	3.2	3.2	3.0
Native flora	As	Cy	Poa	Ra	Ro	Pol	Ca	La	Fa	Sc
	10.3	9.6	8.4	5.0	4.5	3.3	3.0	2.8	2.5	2.4

Note: As—Asteraceae; Cy—Cyperaceae; Poa—Poaceae; Ra—Ranunculaceae; Ro—Rosaceae; Pol—Polygonaceae; Br—Brassicaceae; Ca—Cariophyllaceae; La—Lamiaceae; Fa—Fabaceae; Sc—Scrophulariaceae; Che—Chenopodiaceae; Bo—Boraginaceae.

**Table 2:** Structure of the head genera spectrum.

Vascular Plant Flora	Genus Rank									
	1	2	3	4	5	6	7	8	9	10
		Rank 2–3		Rank 4–5		Rank 6–7			Rank 9–10	
	Car	Ar	Sal	Vi	Sax	Poa	Sau	Pot	Ju	Cal
Number of species	125	32	32	23	23	21	21	20	19	19
Percentage of flora	7.0	1.8	1.8	1.3	1.3	1.2	1.2	1.1	1.0	1.0

Note: Car—Carex; Ar—Artemisia; Sal—Salix; Vi—Viola; Sax—Saxifraga; Sau—Saussurea; Pot—Potentilla; Ju—Juncus; Cal—Calamagrostis.

## Data Availability

The data presented in this study are available on request from the corresponding author.
